# The role of ethylene carbonate (EC) and tetramethylene sulfone (SL) in the dissolution of transition metals from lithium-ion cathodes†

**DOI:** 10.1039/d3ra02535g

**Published:** 2023-07-10

**Authors:** Yonas Tesfamhret, Haidong Liu, Erik J. Berg, Reza Younesi

**Affiliations:** a Department of Chemistry, Ångström Laboratory, Uppsala University Box 538 SE-75121 Uppsala Sweden Yonas.tesfamhret@kemi.uu.se Rreza.younesi@kemi.uu.se

## Abstract

Transition metal (TM) dissolution is a direct consequence of cathode–electrolyte interaction, having implications not only for the loss of redox-active material from the cathode but also for the alteration of solid electrolyte interphase (SEI) composition and stability at the counter electrode. It has widely been reported that the limited anodic stability of typical carbonate-based electrolytes, specifically ethylene carbonate (EC)-based electrolytes, makes high-voltage cathode performance problematic. Hence, the more anodically stable tetramethylene sulfone (SL) has herein been utilized as a co-solvent and a substitute for EC in combination with diethyl carbonate (DEC) to investigate the TM dissolution behavior of LiN_0.8_C_0.17_Al_0.03_ (NCA) and LiMn_2_O_4_ (LMO). EC|DEC and SL|DEC solvents in combination with either LiPF_6_ or LiBOB salts have been evaluated, with LFP as a counter electrode to eliminate the influence of low potential anodes. Oxidative degradation of EC is shown to propagate HF generation, which is conversely reflected by an increased TM dissolution. Therefore, TM dissolution is accelerated by the acidification of the electrolyte. Although replacing EC with the anodically stable SL reduces HF generation and effectively mitigates TM dissolution, SL containing electrolytes are demonstrated to be less capable of supporting Li-ion transport and thus show lower cycling stability.

## Abstract 2

Transition metal (TM) dissolution, a direct consequence of cathode–electrolyte interactivity, triggers cathode redox active component dissolution as well as modifies solid electrolyte interphase (SEI) on the counter electrode.

## Graphical abstract

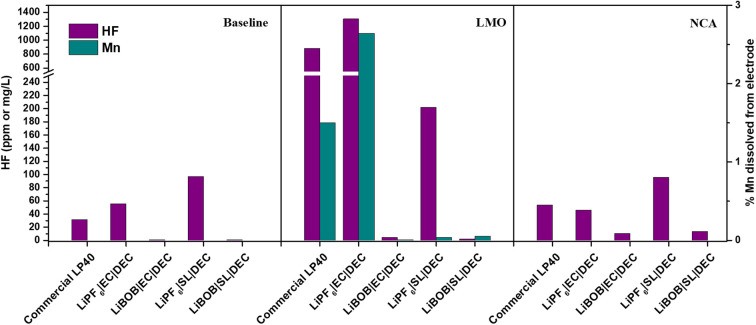

## Introduction

The main development goal for cathode materials is to increase the specific charge *q* as well as the operating potential *E* while maintaining long-term stability. Nickel-rich transition metal (TM) oxides such as NMC811 (LiNi_*x*_Mn_*y*_Co_*z*_O_2_; *x* = 0.8, *x* + *y* + *z* = 1, Ni-rich NMC) and NCA (LiNi_*x*_Co_*y*_Al_*z*_O_2_; *x* = 0.8, *x* + *y* + *z* = 1, Ni-rich NCA) display a relatively high *q* of around 180 mA h g^−1^ in a typical cut-off *E* of 4.2 V *vs.* Li/Li^+^ compared to the well explored cathode materials such as LiCoO_2_ (LCO), spinel-LiMn_2_O_4_ (LMO) and LiFePO_4_ (LFP) which provide a *q* of 145 mA h g^−1^, 120 mA h g^−1^ and 150 mA h g^−1^, respectively.^1–3^ Ni-rich NMC cathodes can however further extract significantly higher *q* when *E* is expanded to higher than 4.2 V *vs.* Li/Li^+^.^4^ However, various processes at the cathode/electrolyte interface, including TM dissolution and oxygen evolution, have been linked to instability of the cathodes.^5–8^ Furthermore, the limited anodic stability of conventional electrolytes makes high-voltage performance difficult to achieve.^1,9^ The highly oxidized state of Ni^4+^ formed during Li^+^ deintercalation can be spontaneously reduced to Ni^3+^ and Ni^2+^ under conditions of *E* > 4.2 V *vs.* Li/Li^+^, by gaining electrons from the electrolyte and initiating oxidative breakdown at the cathode–electrolyte interphase.^6,10^ Release of reactive oxygen singlets from the TM oxide surface promotes electrolyte breakdown and the formation of leaching agents (such as HF), which participate in the dissolution of the remaining undercoordinated surface TM.^6^ This contributes to the structural degradation of the cathode surface. In accordance with existing literature, we recently reported a considerably increased TM dissolution from TM-oxide cathodes in LiPF_6_ based electrolyte compared to non-fluorinated salt based electrolytes, confirming the presence of HF as a main cause of TM dissolution in the form of TMF_*x*_.^11,12^ The LiPF_6_ salt suffers from degradation upon exposure to traces of H_2_O in the electrolyte, which in turn initiates an autocatalytic LiPF_6_ decomposition pathway forming more HF, LiF and POF_3_.^13,14^ The dissolved TM have been shown to diffuse through the separator and be reduced on the anode surface, where it then catalyzes electrolyte side-reactions, increasing interfacial resistance and active lithium loss.^15–17^ Furthermore, at these desired higher *E*, crack formation and oxygen evolution induced by internal stress of cathode surface result in freshly exposed surfaces, providing new sites for cathode–electrolyte interphase reactions and accelerating transition-metal dissolution and overall deterioration of cyclability.^18^ Salt decomposition and TM dissolution are also frequently reported to be accelerated at high *E* by oxidation of organic electrolyte components. Organic Li-ion battery (LIB) electrolytes, particularly those containing ethylene carbonate (EC) co-solvent have been observed to be problematic at high voltages due to considerable gas generation and impedance rise when EC is oxidized.^19^ At *E* greater than 4.3 V, EC molecules dehydrogenate and form dehydrogenated EC (de-H EC), due to the cathode surface oxygen, according to Jung *et al.*^6^ and Zhang *et al.*^20^ The dehydrogenation of EC generates protic species on the surface, which can then trigger reactions with the commonly used LiPF_6_ salt to form HF, transition metal fluorides (TMF) and PF_3_O.^20,21^ EC has long been necessary in organic liquid electrolytes for LIBs, due to it has a high dielectric constant, which is essential for dissociating Li salts in solution. EC further provides an efficient solid electrolyte interphase (SEI) on graphite electrodes.^22–24^ Thus, understanding the contribution of EC to the TM dissolution-based aging mechanism is crucial given that both thermal and electrode potential-activated mechanisms induce TM dissolution.

Several strategies have been explored, including thin film surface coatings, using electrolyte additives, and novel solvent systems, to allow LIB cathodes operate at higher *E* without compromising their stability. Thin Al_2_O_3_, AlF_3_ or ZrO_2_-coatings have been shown to increase rate capability, cycle stability, and interface stability in NMC111/Li half cells operating at 4.5 V *vs.* Li/Li^+^, according to Zou *et al.*, Sun *et al.*, Hu *et al.* and many more.^25–27^ High-voltage NMC/graphite cells have also been shown to better operate with suitable electrolyte additives such as prop-1-ene-1,3-sultone (PES), pyridine boron trifluoride (PBF), 2-aminoethyldiphenyl borate (AEDB), lithium difluoro phosphate (LiDFP) and polyfluoroalkyls(4-(perfluorooctyl)-1,3-dioxolan-2-one, PFO-EC).^17,28–33^ It is frequently expected that novel solvent blends would have higher oxidation *E* than alkyl carbonates (*i.e.* >5 V *vs.* Li/Li^+^). Fluorinated compounds, phosphates and sulfones are common electrolyte components of the majority of recommended solvent blends.^30,31,34,35^ These novel electrolyte systems, however, have also been shown to suffer from poor wettability of common separators, high viscosity and high impedance, as well as substantial cost and safety concerns (*e.g.* fluorinated solvents).^36,37^ Although it also exhibits high viscosity, sulfolane (tetramethylene sulfone, SL) is a good candidate for substituting EC from the electrolyte solution since the oxygen in the sulfolane group can coordinate to the Li^+^ and therefore provide a favorable condition for dissolution of lithium salts.^38,39^ The absence of an O–H group in the structure maybe also render SL less susceptible to the aforementioned dehydrogenation process. SL is known for its potential to produce Li-ion electrolytes with high anodic stability. It has been reported that SL is stable up to 5.5 V *vs.* Li/Li^+^, and that it polymerizes during oxidation, passivating the positive electrode surface.^35^ Furthermore, the electrochemical properties of a LiBOB|SL|diethyl carbonate (DEC) electrolyte have been demonstrated to have high oxidation *E* (>5.3 V) and acceptable conductivity values.^40^ The benefits of LiBOB for cathode surface passivation comprising borate oxalates during oxidation of LiBOB on the cathode surface have also been highlighted by Xu *et al.*^41^ This presents an intriguing opportunity for investigating the electrochemical performance and consequent influence on TM dissolution of the co-solvent systems of SL and DEC with LiBOB and LiPF_6_ salts. However, evaluating the effect of SL on transition metal dissolution from the cathode with graphite, Li-metal, or other low *E* anode materials as counter electrode is challenging and sometimes even misleading due to the effects of TM reduction and deposition on the anode.^42^ Therefore, substituting an anode operating at low *E* with LFP (with high average *E* ∼3.45 V) as the counter electrode avoids the adverse effects of dissolved TM on anode SEI stability. An equal basis for comparing the two co-solvents (EC and SL) is established, and the investigation is focused on the co-solvents' cathode–electrolyte interaction. Herein, we look at the TM dissolution behavior of LiN_0.8_C_0.17_A_0.03_ and spinel LMO by substituting EC with the previously described anodically stable SL as a co-solvent in a mixture with DEC. Furthermore, both LiPF_6_ and LiBOB salts are investigated to observe how these contrast in terms of cycling stability and extent of TM dissolution.

## Results and discussion

### Electrolyte characterization

**Fig. 1:**
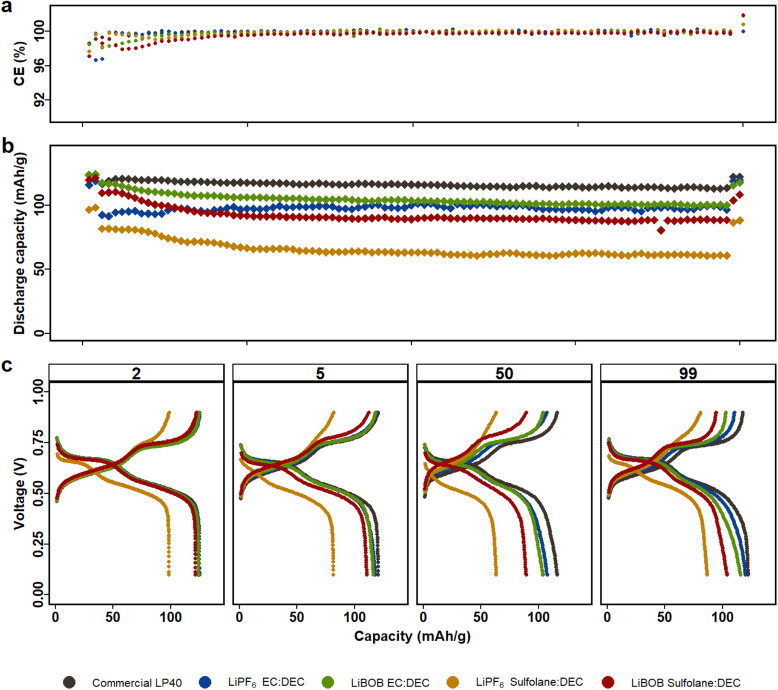
LMO|LFP cells with LiBOB and LiPF_6_ electrolytes in either EC|DEC or SL|DEC solvents are cycled for 100 cycles between 0.2–0.9 V (equivalent to 3.5 and 4.3 V *vs.* Li/Li^+^), first and last two cycles at C/10 and the rest at 1C. (a) Coulombic efficiency. (b) Capacity retention. (c) Voltage profile of the 2nd at C/10, 5th at 1C, 50th at 1C and 99th at C/10 cycles.

The ionic conductivity, H_2_O concentration from coulometric Karl Fischer (CKF) measurements, and HF content of the electrolytes studied here are provided in Fig. S1.† The ionic conductivity is consistently higher in electrolytes based on EC than in those based on SL, at temperatures spanning from 30 °C to 60 °C. Similarly, LiPF_6_ containing electrolytes exhibit higher ionic conductivity than LiBOB containing electrolytes. At 30 °C, the ionic conductivity values for LiBOB|SL|DEC, LiPF_6_|SL|DEC, LiBOB|EC|DEC, and LiPF_6_|EC|DEC are 2.9 mS cm^−2^, 3.5 mS cm^−2^, 5 mS cm^−2^, and 7.3 mS cm^−2^, respectively. LP40 electrolyte has an expected ionic conductivity of 9.3 mS cm^−2^ at 30 °C.^43^ The discrepancy between the prepared LiPF_6_|EC|DEC electrolyte and LP40 electrolyte is solely due to salt concentration, where all prepared electrolytes have a lower salt concentration of 0.7 M. The decision to include 0.7 M salt in the electrolytes is based on the maximum solubility of LiBOB in SL|DEC solvent. Therefore, the salt concentration in the prepared electrolytes is maintained the same. The viscous and lesser ionically conductive SL-based electrolytes should likely suffer in terms of high-rate capability and increased polarization during electrochemical characterization. The absence of HF is, however, naturally favorable for the LiBOB containing electrolytes. As illustrated in Fig. 1, HF can only be detected in electrolytes containing LiPF_6_. The LP40 and LiPF_6_|EC|DEC electrolytes have a higher HF content of 92 mg L^−1^ and 116 mg L^−1^, respectively, than the LiPF_6_|SL|DEC electrolyte with 78 mg L^−1^ of HF. This implies that the presence of H_2_O affects not only the salt, but also the solvents (*e.g.*, EC is susceptible to hydrolysis), resulting in an increased HF after electrolyte preparation.^44^ According to CKF titrations, the water content in the SL, DEC, and EC co-solvents is less than 20 mg L^−1^. H_2_O concentrations in LiPF_6_ containing electrolytes, on the other hand, rise to a comparable ∼60–90 ppm after adding LiPF_6_. Due to the reason that LiBOB reacts with and polymerizes the CKF reagent solution, the water concentration in LiBOB based electrolytes could not be determined, but the 120 °C drying temperature should ensure water content to lie in similar ranges as for the LiPF_6_-based electrolytes.

### Electrochemical characterization

Fig. 1 displays electrochemical performance of LMO|LFP cycled for 100 cycles using the five aforementioned electrolytes in the voltage window of 0.2 V and 0.9 V (corresponding to 3.5 V and 4.3 V *vs.* Li/Li^+^, see Fig. S2†). Similarly, NCA|LFP cell were cycled in full cell voltage window of −0.4 V and 1.1 V (corresponding to 3 V and 4.5 V *vs.* Li/Li^+^), see Fig. S3.† As Kang Xu *et al.* remarked, one key disadvantage of SL is the inability to form protective SEI on graphite anodes.^43^ Our previous study in half cell setup also demonstrated that dissolution of TMs in the electrolyte results in deposition on the surface of the Li-metal, decreasing the chance of ICP quantification of TM in the electrolyte.^45^ Therefore, LFP is selected as the counter electrode to restrict the reduction of Mn-ions on the anode and counter electrode's contribution to overall cell performance. The LFP anodes had previously undergone a full delithiation/lithiation cycle, followed by delithiation up to 85%. The full cell anode to cathode capacity ratio is 62 : 38. (2.9 mA h : 1.8 mA h). The LFP counter electrode is restricted to a flat potential plateau (3.45 V), but the relative LMO or NCA working electrode potential varies during galvanostatic cycling, hence preventing any potential full cell voltage slippage with respect to LFP. The difference in voltage profiles between the first two formation cycles and the rest demonstrates capacity loss due to an increase in C-rate. The first two cycles are at C/10, and the remaining 96 cycles at 1C. The cycling program is finalized, with the last two cycles at C/10, completing 100 cycles to evaluate capacity loss.

All LMO|LFP cells feature two plateaus, which can be attributed to two-stage intercalation/deintercalation process of spinel LMO. With a slightly higher capacity over 100 cycles and a lower overpotential of 70 mV at C/10 and 110 mV at 1C, LP40 exceeds the other electrolytes in performance (Fig. S4a†). This is expected given that LP40 in comparison to the prepared LiPF_6_|EC|DEC (i) contains higher salt concentration (1 M) in comparison to the prepared (0.7 M) and (ii) contains lower impurities in terms of H_2_O and HF from start. The initial discharge capacities of the prepared electrolytes decrease in the order of LiPF_6_|EC|DEC, LiBOB|EC|DEC, LiBOB|SL|DEC and LiPF_6_|SL|DEC, with respective capacities of 125 mA h g^−1^, 124 mA h g^−1^, 120 mA h g^−1^, and 97 mA h g^−1^ (at C/10). After 100 cycles, the discharge capacity decreases marginally to 118 mA h g^−1^, 118 mA h g^−1^, 110 mA h g^−1^ and 82 mA h g^−1^ at 1C for the respective electrolytes, in comparison to LP40, which has a higher capacity of 121 mA h g^−1^ at the same C-rate (Fig. 1b). Long-term cycle stability demonstrates that SL-containing cells do have larger overpotential than EC-containing cells, which is also reflected in capacity fade. The capacity loss for the EC-based electrolytes of LiPF_6_ and LiBOB is 3.5% and 4.9%, respectively, compared to 4.4% and 9.6% for the SL-based electrolytes of the same salts. Although the LiPF_6_|EC|DEC cells, both commercial and prepared, outperform the LiBOB|EC|DEC cell, the contrary is true for SL containing cells, where LiBOB based electrolyte is more stable than LiPF_6_.

**Fig. 2:**
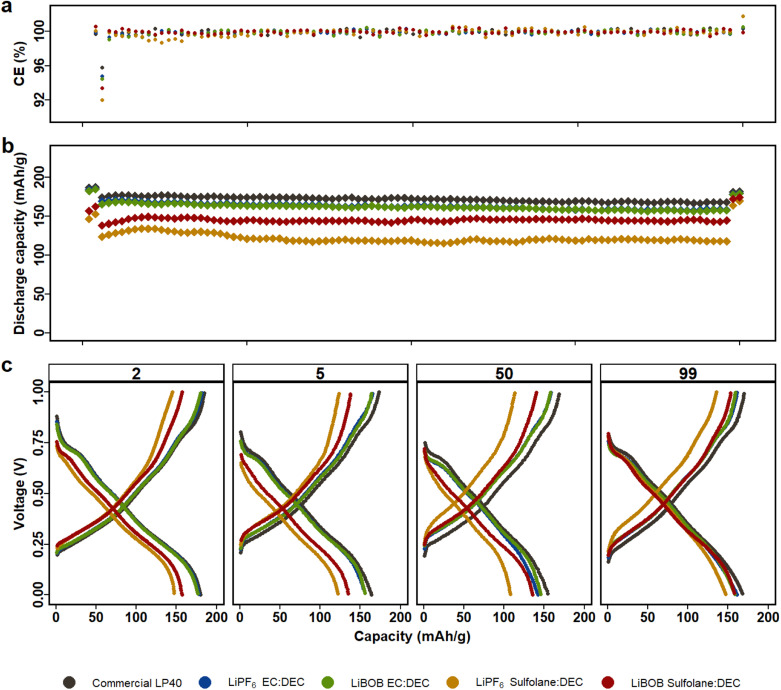
NCA|LFP cells with LiBOB and LiPF_6_ electrolytes in either EC|DEC or SL|DEC solvents are cycled for 100 cycles between the voltage window of 0.2–0.9 V (equivalent to 3.5 and 4.5 V *vs.* Li/Li^+^), first and last two cycles at C/10 and the rest at 1C. (a) Coulombic efficiency. (b) Capacity retention. (c) Voltage profile of the 2nd at C/10, 5th at 1C, 50th at 1C and 99th at C/10 cycles.

LP40 outperforms the other electrolytes in NCA|LFP cells (Fig. 2), with increased capacity, superior capacity retention over 100 cycles, and a lower overpotential of 80 mV at C/10 and 120 mV at 1C (Fig. S4b†). This is similar to what we observed above for LMO|LFP cells. The prepared electrolytes show initial capacities in decreasing order of LiPF_6_|EC|DEC, LiBOB|EC|DEC, LiBOB|SL|DEC, and LiPF_6_|SL|DEC, with initial capacities of 187 mA h g^−1^, 182 mA h g^−1^, 157 mA h g^−1^, and 147 mA h g^−1^, respectively, when compared to LP40, which shows a capacity of 187 mA h g^−1^ at the same C-rate. Capacity decreases slightly to 175 mA h g^−1^, 170 mA h g^−1^, 138 mA h g^−1^, and 124 mA h g^−1^ at 1C (starting from the 3rd cycle) for the equivalent electrolytes. SL based electrolytes show higher polarization of ∼200 mV compared to EC-based cells with polarization of ∼150 mV, which is also reflected in capacity fade. In addition to the reduced ionic conductivity observed for the SL-based electrolytes, the cells using EC-based electrolytes indicate that the electrode/electrolyte interphase layer generated is more conductive for Li+ ion transport. In SL-based NCA and LMO cells, LiBOB-containing cells outperform LiPF_6_-based cells in terms of long-term cycling stability. Several studies have reported that combining LiBOB with SL has a favorable synergistic effect, specifically higher oxidative stability.^40,46^ Furthermore, the use of LiBOB as an electrolyte additive for cathode film formation has also been reported on several studies.^47,48^ When the difference in ionic conductivity is not discernable between LiPF_6_|SL|DEC and LiBOB|SL|DEC (see Fig. S1†), the more thermally and electrochemically stable LiBOB conducting salt demonstrates superiority.^49,50^ The comparable cycling performance of LiBOB|EC|DEC with LiPF_6_|EC|DEC in both NCA and LMO cells also supports the observation that, when ionic conductivity is removed from the equation, LiBOB provides a formation of an efficient CEI. LiPF_6_|SL|DEC and LiBOB|SL|DEC both show capacity increase of 14% and 10% at C/10 in the last two cycles, respectively, following a current rate change and indicating that wettability and electrolyte conductivity issues remain.

### Postmortem characterization

**Fig. 3:**
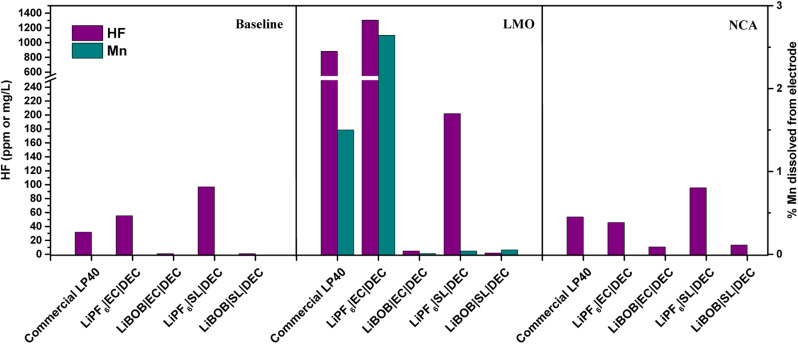
Measured HF in the electrolyte and Mn% dissolved into the electrolyte after 100 cycles. Baseline (uncycled electrolyte).

The electrolytes extracted from cycled LMO|LFP and NCA|LFP cells, as well as electrolytes kept free of active materials for the duration of the experiment (baseline), are analyzed using ICP and F^−^ probe, as shown in Fig. 3. In comparison to the electrolytes after preparation, there is a decrease in HF concentration for the baseline when the electrolytes are not in contact with active materials (see HF in Fig. 3*vs.* HF Fig. S1†). HF, which has a high autoprotolysis constant (10^−12.5^) and is highly reactive, is consumed in the fresh electrolyte containers before measurement. HF clearly increases in the LMO cells, however, HF content in the NCA cells is the same as the baseline. There is a direct correlation between the presence of LiPF_6_ in the electrolytes and the detection of HF. The prepared LiPF_6_|EC|DEC for LMO has the highest HF concentration at 1304 mg L^−1^, followed by LP40 at 880 mg L^−1^. Despite containing 0.7 M LiPF_6_ rather than 1 M LiPF_6_ in the commercial LP40 electrolyte, the prepared LiPF_6_|EC|DEC electrolyte generates more HF after cycling. This is due to the increased amount of H_2_O impurity, shown in Fig. S1,† of which, due to LiPF_6_ decomposition, has a propensity to generate more HF and thereby also TMF. At 53 mg L^−1^ and 46 mg L^−1^, respectively, the HF concentrations in the cells with LiPF_6_|EC|DEC and LP40 for NCA are comparable. HF is also recorded in cells containing LiPF_6_|SL|DEC, however, at lower levels of 202 mg L^−1^ and 95 mg L^−1^ for LMO and NCA, respectively. LMO|LFP cells generate an order of magnitude higher HF than NCA|LFP cells when both LiPF_6_ and EC are present in the electrolyte. Due to the difference in specific surface area between NCA (0.36 m^2^ g^−1^) and LMO (1.86 m^2^ g^−1^), the electrode surface and electrolyte contact are varied. Thus, the process of EC dehydrogenation, which generates protic species on the cathode material surface and can later initiate interactions with the breakdown of LiPF_6_ salt to form HF, occurs more frequently in LMO cells than in NCA cells.^20^ The relatively unchanged HF concentrations in NCA's electrolytes support the hypothesis that the process is surface area dependent, and the lower surface area NCA does not reflect such an increase in HF concentration. This is also mirrored in the TM dissolution properties of both materials, with LMO|LFP cells demonstrating Mn dissolution only in the case of LiPF_6_|EC|DEC cells. Unexpectedly, Ni or Co dissolved in the electrolyte could not be detected in any of NCA cells. This is consistent with previous research that shows limited Co dissolution in NCA, but it is unexpected for Ni as it accounts for 80% of the TM available in the cathode. The onset of TM dissolution in Ni-rich layered oxides has been reported to be at *E* > 4.5 V.^5,11,51^ This is due to an increase in acidic species generated from the electrolyte during oxidative degradation. Therefore, TM dissolution for NCA will be limited in the conditions studied here. Al dissolution is difficult to analyze since electrolytes are supplied in Al containers and the electrolyte already contains Al, making it difficult to obtain a precise quantification of the possibly dissolved Al from NCA.

### Discussion

Several autocatalytic mechanisms are involved in the generation of HF during 100 cycles; nevertheless, it is evident from the LMO|LFP cells that the fluorinated salt of LiPF_6_ significantly contributes to the TM dissolution process, whereas LiBOB did not. The phenomenon of LiPF_6_ salt decomposition in the presence of trace water, leading to the formation of hydrofluoric acid (HF), has been extensively reported in literature.^1,2^ The reaction can be succinctly represented as follows:LiPF_6_ + H_2_O → LiF + HF + PF_5_

Further interaction of PF_5_ with water results in additional HF:PF_5_ + H_2_O → 2LiF + 2HF + POF_3_

These reactions underline the significant role that trace amounts of H_2_O play in not only influencing the stability of the salt but also predisposing the solvents to decomposition. Notably, ethylene carbonate (EC) exhibits a tendency towards hydrolysis. The resultant dehydrogenation of EC produces protic species on the electrode surface, which can induce further reactions with LiPF_6_. This reaction cascade ultimately leads to the formation of HF, transition metal fluorides (TMF), and PF_3_O.^20,21^ In LMO|LFP cells, the 2.6% and 1.5% capacity losses associated with Mn loss in LiPF_6_|EC|DEC and LP40 electrolytes, respectively, are slightly lower than the 3.5% and 1.8% capacity losses associated with 100 times cycling. This implies that capacity loss is caused not only by the loss of the redox active component of the cathode, but also by its subsequent influence on the electrode, such as increased interfacial resistance after cathode surface deterioration. The dissolution of Mn from LMO, on the other hand, has been established to be the outcome of (i) Jahn–Teller distortion and (ii) acid–base interaction between the alkaline electrode surface and acid electrolyte environment.^52,53^ Mn^3+^ triggers the Jahn–Teller phenomenon, which generates an octahedra complex symmetry distortion and transforms the spinel to a tetragonal symmetry. At high current rates, this process becomes more apparent as Li-diffusion in the electrolyte is much faster than in the LMO structure, resulting in more Li^+^ concentrating at the surface of LMO particles and forming a Mn^3+^ rich electrode surface. It is widely understood that the disproportionation reaction of Mn^3+^ results in the dissolution of Mn^2+^ into the electrolyte.^54^

**Fig. 4:**
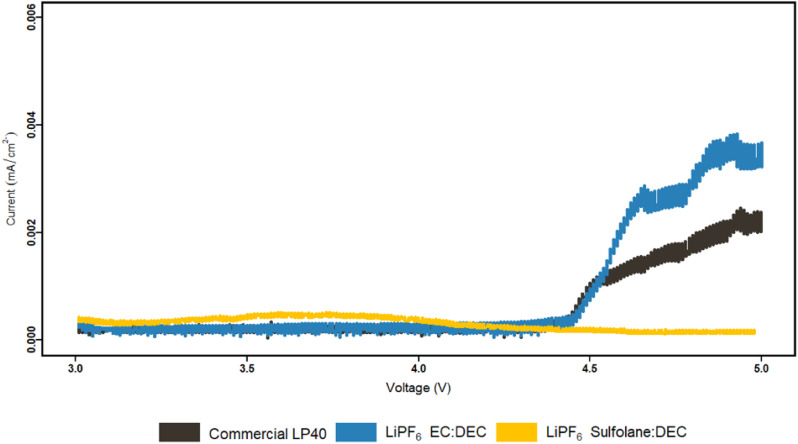
PITT characterization of LP40, LiPF_6_|EC|DEC and LiPF_6_|SL|DEC electrolytes. Carbon coated Al electrodes were cycled up to 5 V *vs.* Li/Li^+^.

Furthermore, our prior study showed that spinel LMO with lower alkalinity than its layered oxide (NCA) counterpart has a lesser tendency for neutralizing the acidic electrolyte surroundings, resulting in an easier environment for TM dissolution.^53^ We addressed the chemical dissolution profiles of LMO and NMC, demonstrating that HF concentration decreases once the electrolyte encounters the active materials due to a thermal acid–base interaction. In contrast, for LMO here, it is shown that the HF increases in contact with the active material under electrochemical cycling conditions (Fig. 3). This implies the formation of HF *via* electrochemical contributions, principally EC based. Since EC-based cycled electrolyte for LMO demonstrates a much sharper increase in HF concentration, the electrochemical stability of EC can be called into doubt. This is supported by PITT measurements comparing LiPF_6_|EC|DEC and LiPF_6_|SL|DEC electrolytes to LP40 (Fig. 4). The EC-containing electrolyte begins to pass charge slightly below 4.4 V *vs.* Li/Li^+^, whereas the SL-based electrolyte appears to be stable up to 5 V *vs.* Li/Li^+^. However, this is not reflected in the cycling performance of SL-based electrolytes, which show larger overpotentials, lower initial capacities, and faster capacity fading. This emphasizes the importance of increased ionic conductivity and, to a lesser degree, the cathode electrolyte interphase (CEI) layer, which EC-based electrolytes appear to be superior in, whereas SL-based electrolytes rely on the inclusion of additives for interfacial stability in most studies.^48,55,56^ The (i) lower dissociation degree of LiBOB (0.65), compared to LiPF_6_ (0.7), and high viscosity of SL result in a low Li^+^ diffusion coefficient of the cathodes, hence lowering the rate capability of the cells. The rate of Li^+^ intercalation/de-intercalation during charge/discharge is influenced by Li^+^ transport at electrode/electrolyte interphase. CEI should be homogeneous, structurally stable, promote Li^+^ transport, prevent an interface reaction between the electrolyte and the electrode and improve electrode reaction rate.^56^ SL-based electrolyte, despite adhering to the structural characteristics at high voltages by minimizing further HF formation and restricting TM dissolution, suffers in supporting Li^+^ transport at high cycling rates. Although, EC-based electrolytes degrade chemically and electrochemically at higher potentials, they are superior to SL-based electrolytes studied here. However, HF and its stimulators LiPF_6_ and H_2_O are the principal culprits in TM dissolution. Eliminating fluorinated salt, introducing HF scavenging additives, or introducing film-forming additives to improve the properties of CEI, which can allow Li^+^ migration, can dramatically reduce TM dissolutions and improve cycling stability.

## Experimental

### Cell materials

Slurry containing cathode active materials (90 wt%), carbon black (Imerys, C65, 5 wt%), and PVdF binder (5 wt%, PVdF-HFP, Kynar Flex 2801) in *N*-methyl-2-pyrrolidone (NMP, VWR) was homogenized for 1 hour in Retch MM 4400 ball miller (25 Hz). Binder was previously prepared as a 7 wt% PVdF and 94 wt% NMP solution. The liquid content of LMO (SEDEMA), NCA (Helium tech), and LFP (LIFE POWER P2) slurries was 60 wt%, 61 wt%, and 53 wt%, respectively. NCA composition is determined by ICP-OES to be LiN_0.8_C_0.17_A_0.03_ (Table S1†). The slurries were then cast on a carbon coated Al-foil (20 μm thickness), with a coating gap of 150 μm specified on the film applicator. For LMO, NCA, and LFP, the fabricated electrode coatings have practical capacities of 1 ± 0.06 mA h cm^−2^ (∼6 mg cm^−2^), 1.2 ± 0.05 mA h cm^−2^ (∼6 mg cm^−2^) and 0.9 ± 0.1 mA h cm^−2^ (∼8.3 mg cm^−2^), respectively. The capacities are calculated based on the 120 mA h g^−1^, 195 mA h g^−1^, and 150 mA h g^−1^ practical capacities of LMO, NCA, and LFP. The coatings were dried in a vacuum oven at 70 °C before being perforated into electrodes of 15 mm, 13 mm, and 12 mm diameters for LFP, LMO, and NCA. Before use, all electrodes were dried under vacuum at 120 °C for 12 hours in an Ar-filled glovebox glove box (O_2_ < 1 ppm, H_2_O < 1 ppm).

### Cell assembly

LMO|LFP and NCA|LFP cells with N/P ratio of ∼1.1 were assembled in a pouch bag format with four separators, two of which are Celgard (2325, 33 mm) and two of which are glass fiber (Whatman, grade GF/A, 20 mm diameter). Celgard and glass fiber separators were vacuum dried for 24 h at temperatures of 70 °C and 120 °C, respectively. All electrodes were connected with aluminum current collectors (25 μm, thickness). The LFP electrodes underwent a complete delithiation/lithiation cycle, followed by delithiation up to 85% in a separate pouch (Li|LFP) containing a 22 mm diameter Li-metal counter electrode, a 3 × 3 cm Celgard (2325), and 100 μL of the equivalent electrolyte that will be used in the full cell. Potentiometric intermittent titration technique (PITT) cells were assembled in pouch bag format with carbon coated Al (13 mm) and Li-metal (15 mm) electrodes, separated by Celgard.

### Electrochemical characterization

All galvanostatic cycling tests were carried out at room temperature (20–22 °C) using a LANDT battery testing equipment (model CT2001 A). Prior to cycling, the cells were maintained at OCV for 5 h. The initial two cycles were performed at C/10, followed by 96 cycles at 1C, and the cycling program concluded with the final two cycles at C/10, completing 100 cycles. C-rates were estimated using the 120 mA h g^−1^, 190 mA h g^−1^, and 150 mA h g^−1^ practical capacities of LMO, NCA, and LFP, respectively. LMO|LFP cells were cycled between 0.2 V and 0.9 V cell voltage, which corresponds to 3.4 and 4.3 V *vs.* Li/Li^+^. NCA|LFP cells were cycled between −0.2 V and 1.1 V, corresponding to 3.2 and 4.5 V *vs.* Li/Li^+^. Li|LFP cells were cycled between cell voltages of 3 V and 4.1 V for LFP delithiation. Arbin laboratory battery cycling system and Biologic was utilized for three-electrode cells and PITT, respectively.

### Conductivity measurements

A Mettler Toledo SevenGo Duo pro Conductivity meter with an InLab 738ISM probe was used to measure conductivity. The measurements of conductivity were made in an Ar filled glove box (O_2_ < 1 ppm, H_2_O < 1 ppm). The conductivity of the electrolytes was monitored continuously while they were gradually warmed together in an aluminum block to improve heat dispersion from the heating plate.

### CKF

Coulometric Karl Fischer (CKF) titration technique was used to determine the water content in the electrolytes. For measurement, a Metrohm 756 CKF Coulometer was utilized, with roughly 1 gram of electrolyte injected into CKF reagent solution. The test was carried out under typical atmospheric circumstances. As a result, the samples were all exposed to the environment for <10 seconds before being measured.

### ICP

The electrolyte solvents were made from a 1 : 1 vol% mixture of tetramethylene sulfone (sulfolane, SL, Sigma Aldrich) and diethyl carbonate or a 1 : 1 vol% mixture of ethylene carbonate (EC, BASF) and diethylene carbonate (DEC, GOTION). Before adding salts, the solvents were dried for a week with molecular sieves. Each cell's electrolyte volume was set to 300 μL. Lithium bis(oxalato)borate (LiBOB, Solvionics, 99.9%) and lithium hexafluorophosphate (LiPF_6_, Solvionics, 99.9%) were dried at 80 °C for 48 hours under vacuum, then added to the prepared solvents to form (i) 0.7 M LiPF_6_|EC|DEC (ii) 0.7 M LiPF_6_|SL|DEC (iii) 0.7 M LiBOB|EC|DEC (iv) 0.7 M LiBOB|SL|DEC. Before filtration, molecular sieves were added to the prepared electrolytes for 72 hours. LP40 (1 M LiPF_6_|EC|DEC, Solvionics) was also prepared as is for reference.

The glass fiber separators, with a thickness of 1.6 μm, can retain enough electrolyte to be extracted after cycling and analysis by ICP-OES. During extraction of the post-cycled electrolyte, the outer Celgard separators prevent against contamination from the working and counter electrodes. The extracted glass fiber separators with post-cycled electrolyte were placed in 2 mL epindorf centrifugation tubes (VWR) and centrifuged for 10 minutes at 2000 rpm. Because the glass fibers were attached to the top of the epindorf, the electrolyte was drained to the bottom during centrifugation. For each cell, 100 μL of extracted electrolyte was sampled for ICP-OES analysis.

The electrolyte samples were evaporated by heating at 250 °C for 16 hours before diluting with nitric acid (HNO_3_, 65%, VWR) and diluting 100× with milli Q water type I water (Fisher Scientific). For ICP measurements, an Avio 500 Scott/Cross-Flow configuration was used. The ICP-OES was calibrated using manganese, nickel, and aluminum concentrations of 1, 0.5, 0.1, and 0.05 g mL^−1^ obtained from the Multi-element Calibration Standard (PerkinElmer). ICP quantification was performed on LMO powder, NCA powder and various electrolytes. The results of dissolved TM quantification in electrolytes are reported as a weight percentage based on the available TM in the LMO and NCA electrodes (the raw data and detail values for ICP-OES measurements are provided in ESI†).

### Fluoride detector

The HF was determined using an F^−^ sensitive ion selective electrode (ISE, Mettler Toledo perfectION combination fluoride electrode). The methodology is based on Strmcnik *et al.*'s method developed for F^−^ determination in the electrolytes.^57^ Prior to measurements, the F^−^ ISE was calibrated with a set of standards having a known quantity of F^−^ ions. The calibration standards were composed of water and used NaF as a fluorine source. Calibration standards with F^−^ concentrations ranging from 0.5 to 500 mg L^−1^ were utilized to create a calibration curve, which was then used to quantify HF in the electrolytes. To compensate for charge contribution from contaminants, a 50 : 50 vol% of water and total ion strength adjustment buffer (TISAB) is utilized as a base.

### BET

The Brunauer–Emmett–Teller (BET) surface area of active materials was determined using nitrogen-gas physisorption (ASAP 2020 analyzer).

## Conclusions

The anodically stable SL has been used as a co-solvent and a substitute for EC in combination with DEC to study the TM dissolution behavior of NCA and spinel LMO. EC|DEC and SL|DEC solvents in combination with either LiPF_6_ or LiBOB salts have been analyzed, where LFP is selected as a counter electrode to eliminate any contribution from low potential anodes. SL-based electrolytes exhibit superior electrochemical stability of up to 5 V *vs.* Li/Li^+^ in PITT measurements, this is evidenced by minimizing electrode potential-based HF evolution and eliminating TM dissolution. However, SL-based electrolytes are unable to support the Li^+^ transfer at higher C-rate, which is mirrored in their lower ionic conductivity and higher polarization when cycled in LMO|LFP and NCA|LFP setup. EC-based electrolytes degrade chemically and electrochemically at higher potentials, which is reflected in the generated HF and TM dissolved into the electrolyte. Nevertheless, EC-based electrolytes outperform SL-based electrolytes in terms of cycle stability and capacity retention (in LMO|LFP and NCA|LFP setup). Furthermore, the amount of dissolved TM does not account for capacity loss following 100 cycles. The main cause of TM dissolution is identified as HF and thereby LiPF_6_, as LiBOB based electrolytes show no sign of TM dissolution. Chemical/electrochemical degradation of EC at the cathode surface accelerates HF generation and emphasizes the cause. The cells with LiPF_6_|EC|DEC, which is the most often utilized electrolyte combination, however showed higher outperforming cycling performance in this study, which indicates that a more desirable CEI is formed. This highlights the importance of CEI compared ageing mechanism in relation to TM dissolution and HF interactions.

## Conflicts of interest

The authors declare that they have no known competing financial interests or personal relationships that could have appeared to influence the work reported in this paper.

## Supplementary Material

RA-013-D3RA02535G-s001

RA-013-D3RA02535G-s002

RA-013-D3RA02535G-s003

RA-013-D3RA02535G-s004

RA-013-D3RA02535G-s005

RA-013-D3RA02535G-s006

RA-013-D3RA02535G-s007
