# Supplementary figures and images for: The role of ethylene carbonate (EC) and tetramethylene sulfone (SL) in the dissolution of transition metals from lithium-ion cathodes
Source: RSC Adv. 2023 Jul 10;13(30):20520–9. doi: 10.1039/d3ra02535g (PMC10331795; doi:10.1039/d3ra02535g)

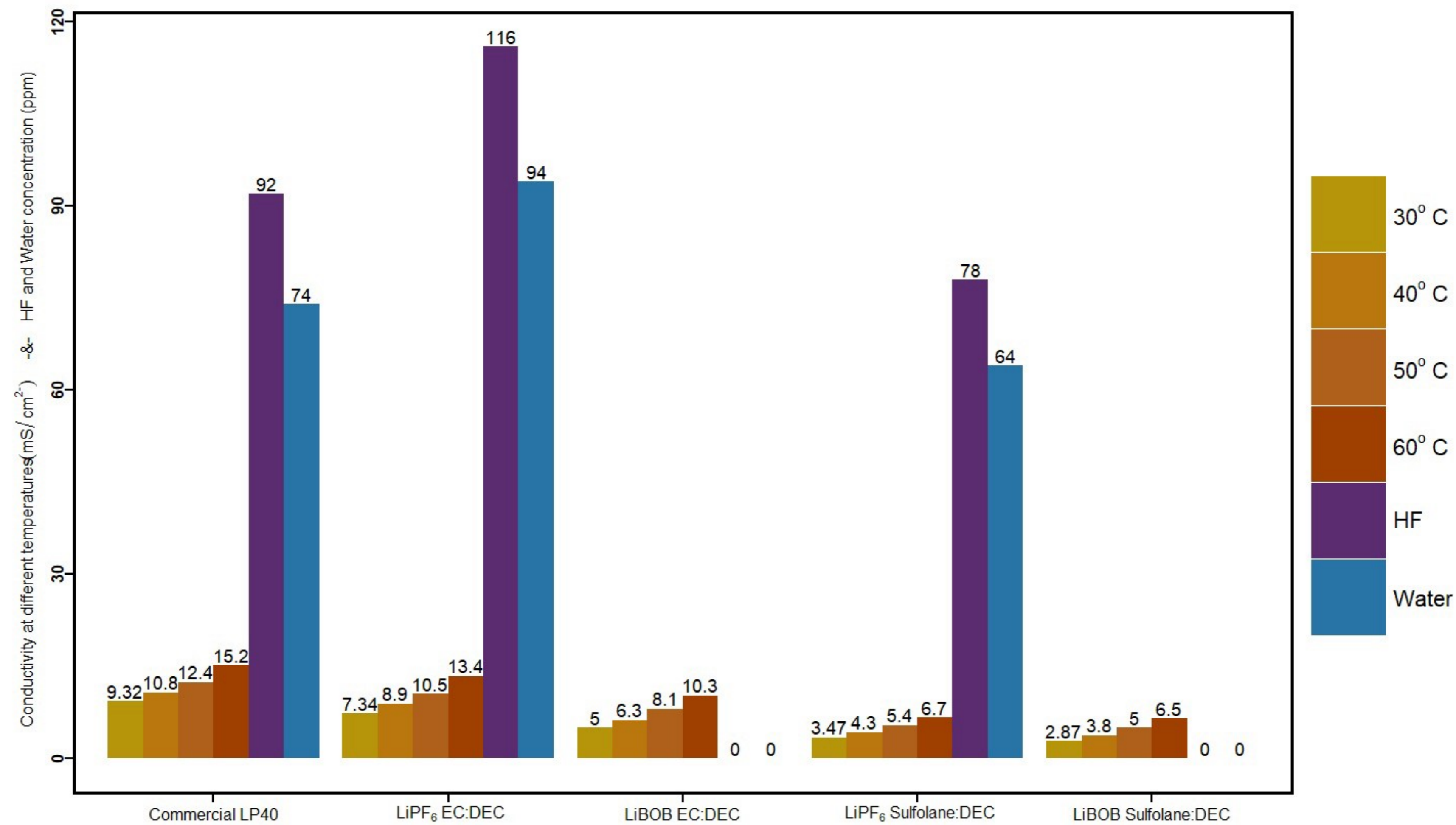

Supplement: RA-013-D3RA02535G-s002 [file RA-013-D3RA02535G-s002.pdf]

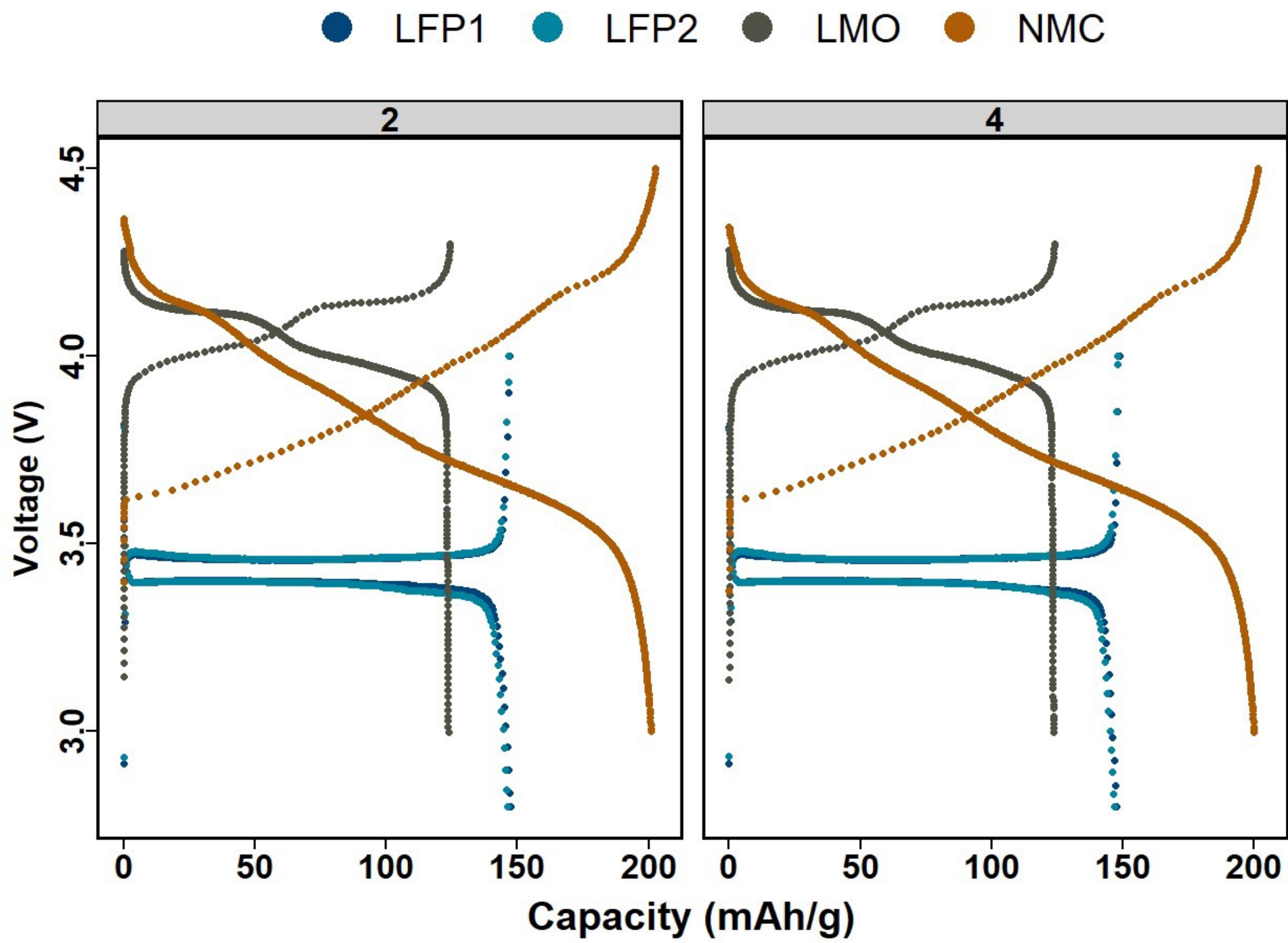

Supplement: RA-013-D3RA02535G-s003 [file RA-013-D3RA02535G-s003.pdf]

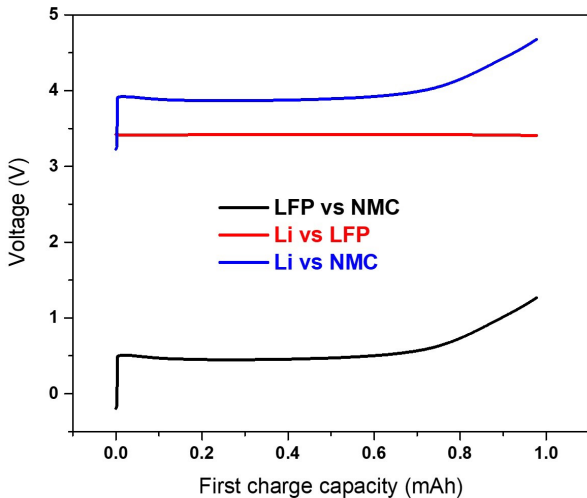

Supplement: RA-013-D3RA02535G-s004 [file RA-013-D3RA02535G-s004.pdf]

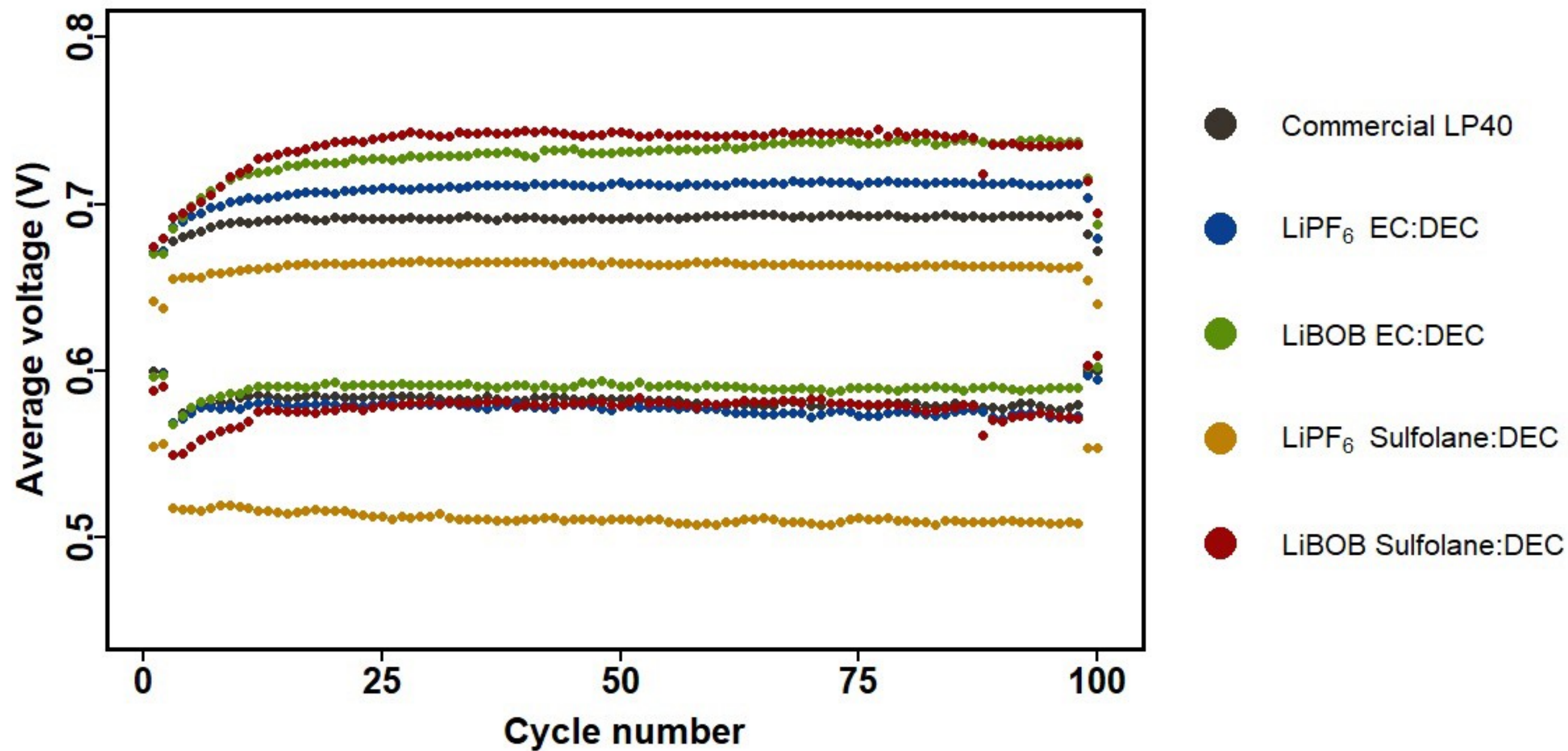

Supplement: RA-013-D3RA02535G-s005 [file RA-013-D3RA02535G-s005.pdf]

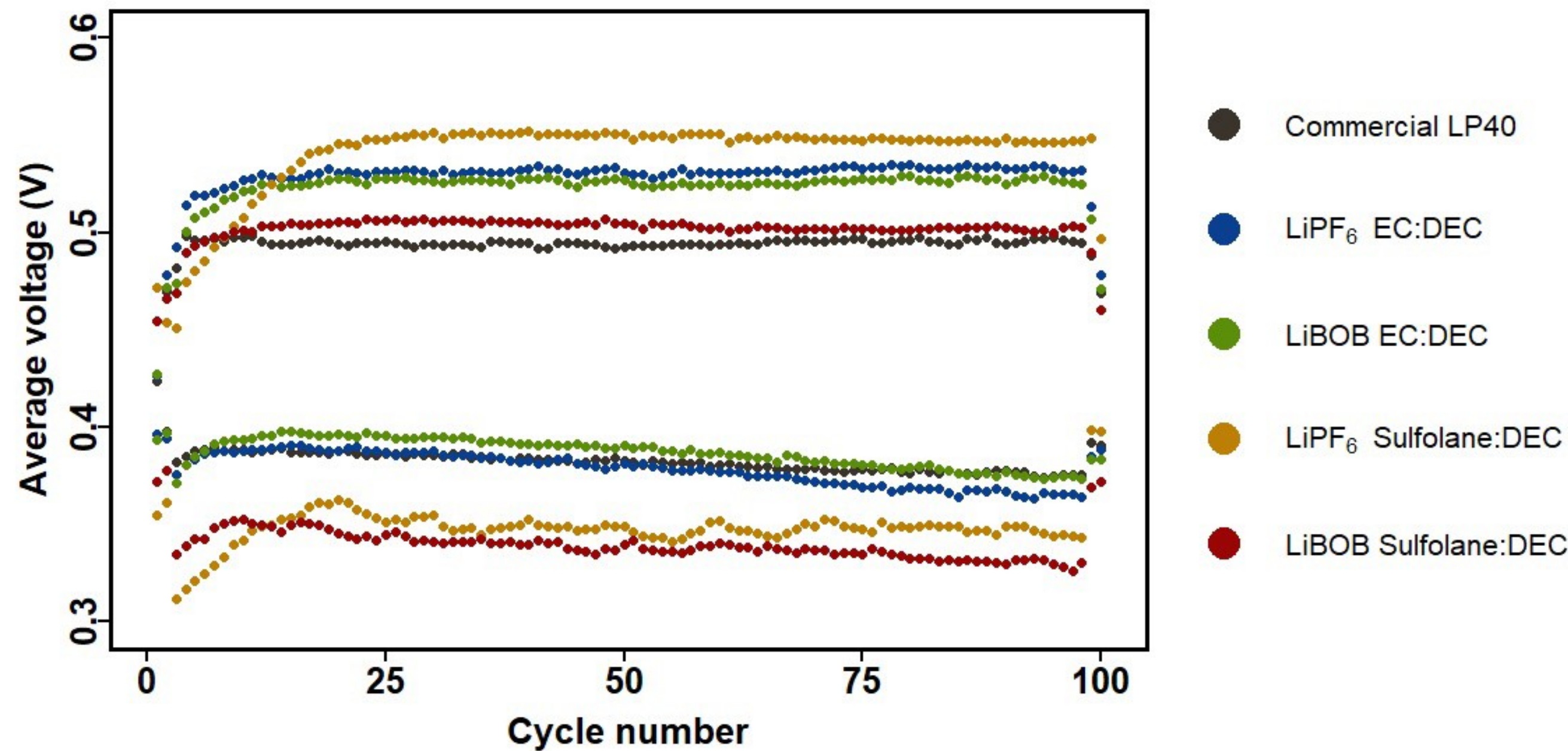

Supplement: RA-013-D3RA02535G-s006 [file RA-013-D3RA02535G-s006.pdf]
