# Supplementary material for: The role of ethylene carbonate (EC) and tetramethylene sulfone (SL) in the dissolution of transition metals from lithium-ion cathodes
Source: RSC Adv. 2023 Jul 10;13(30):20520–9. doi: 10.1039/d3ra02535g (PMC10331795; doi:10.1039/d3ra02535g)
Supplement: RA-013-D3RA02535G-s007 [file RA-013-D3RA02535G-s007.pdf]

Electronic Supplementary Material (ESI) for RSC Advances.
This journal is © The Royal Society of Chemistry 2023

Supplementary informaion

The Role of EC and sulfolane on the dissolution of transition metals from Lithium-Ion Cathodes

Yonas Tesfamhret*[a], Haidong Liu[a], Erik J. Berg[a] and Reza Younesi[a]

[a]Department of Chemistry - Ångström Laboratory, Uppsala University, Box 538, SE-75121 Uppsala, Sweden

* yonas.tesfamhret@kemi.uu.se

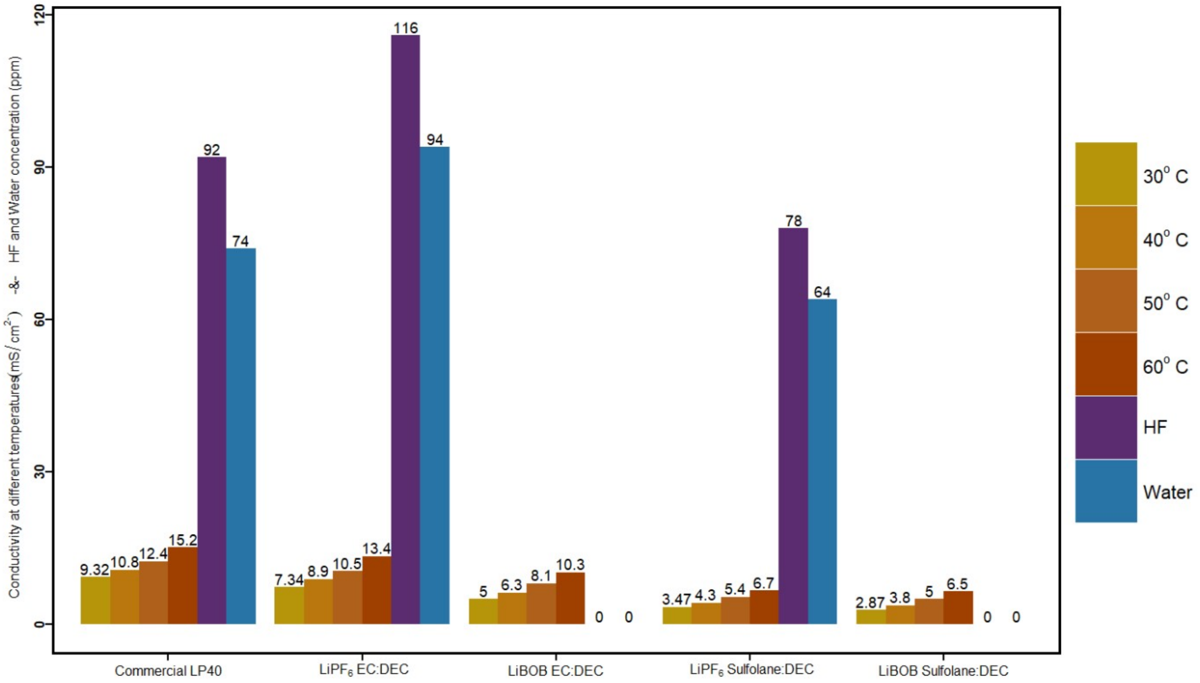

Figure S 1. The ionic conductivity, H_2O and HF concentration of LP40 and prepared electrolytes of 0.7 M LiBOB|SL|DEC, 0.7 M $LiPF_6$|SL|DEC, 0.7 M $LiPF_6$|EC|DEC and 0.7 M LiBOB|EC|DEC. Ionic conductivity is conducted at 30 °C, 40 °C, 50 °C and 60 °C.

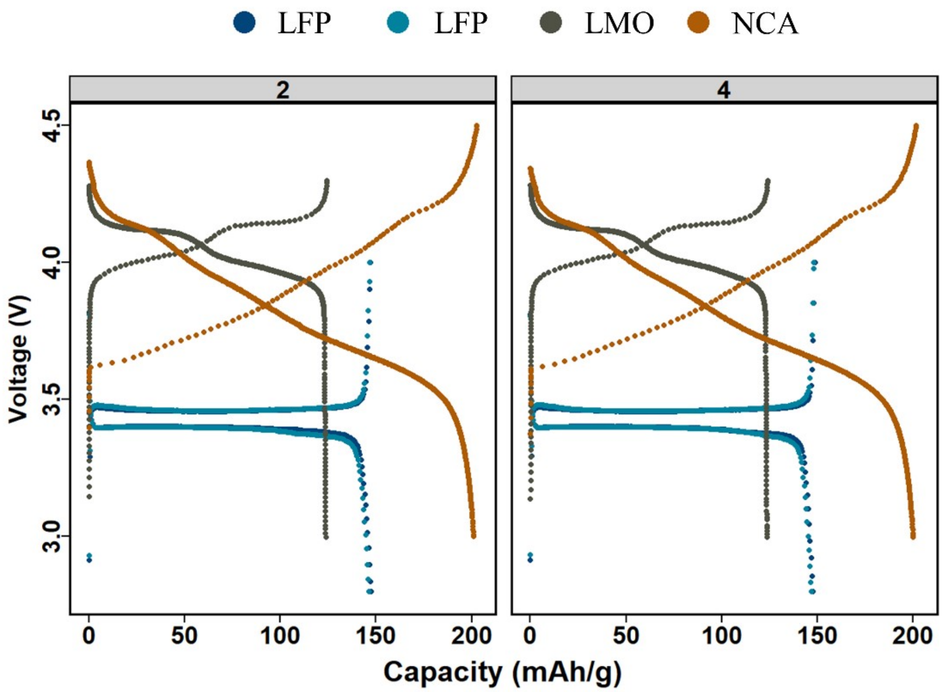

Figure S 2. Half-cell cycling of LFP, LMO and NCA (second and fourth cycle).

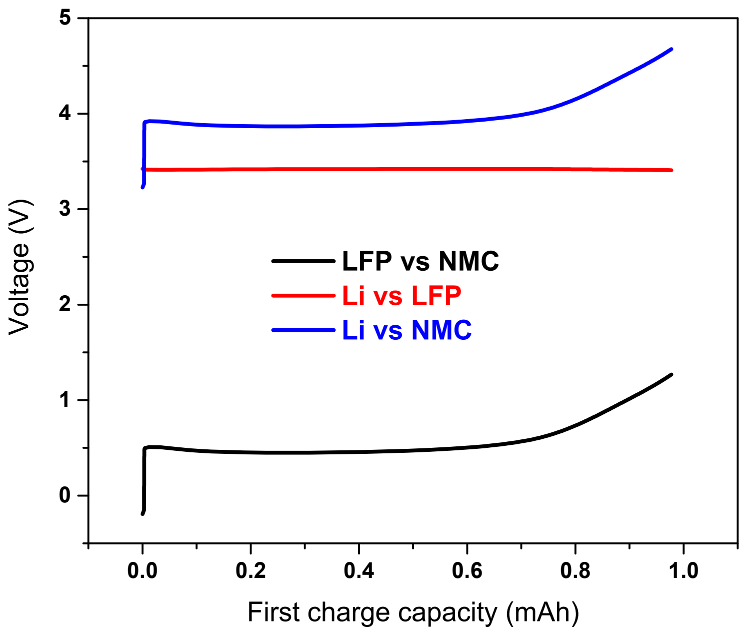

Figure S 3. First charge of a three-electrode cell cycling of LFP|Li|NCA.

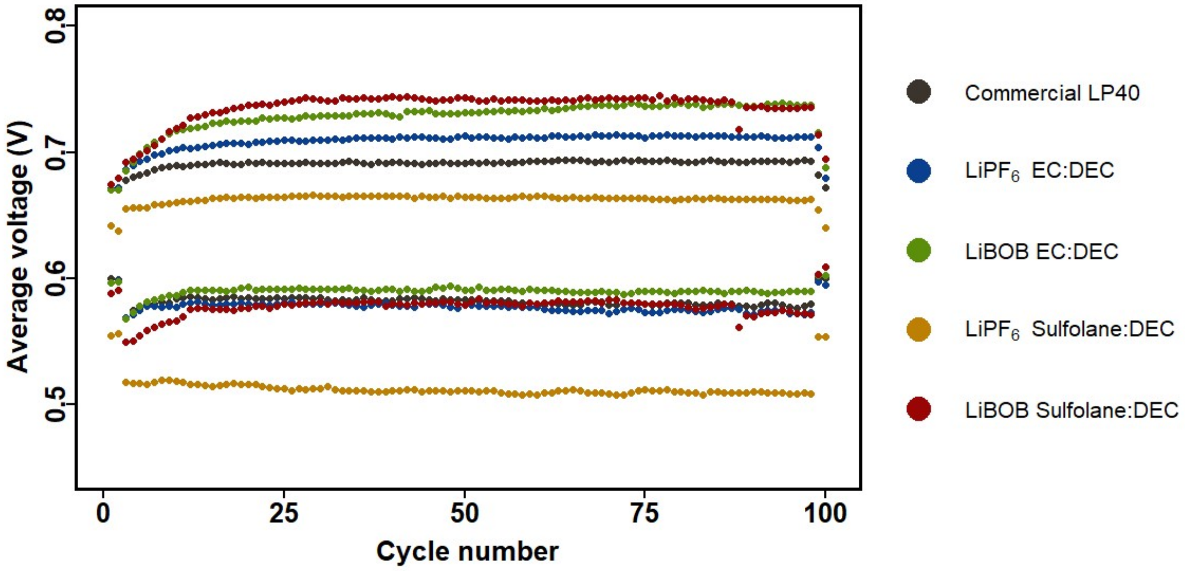

Figure S 4. Average voltage of LMO full-cells.

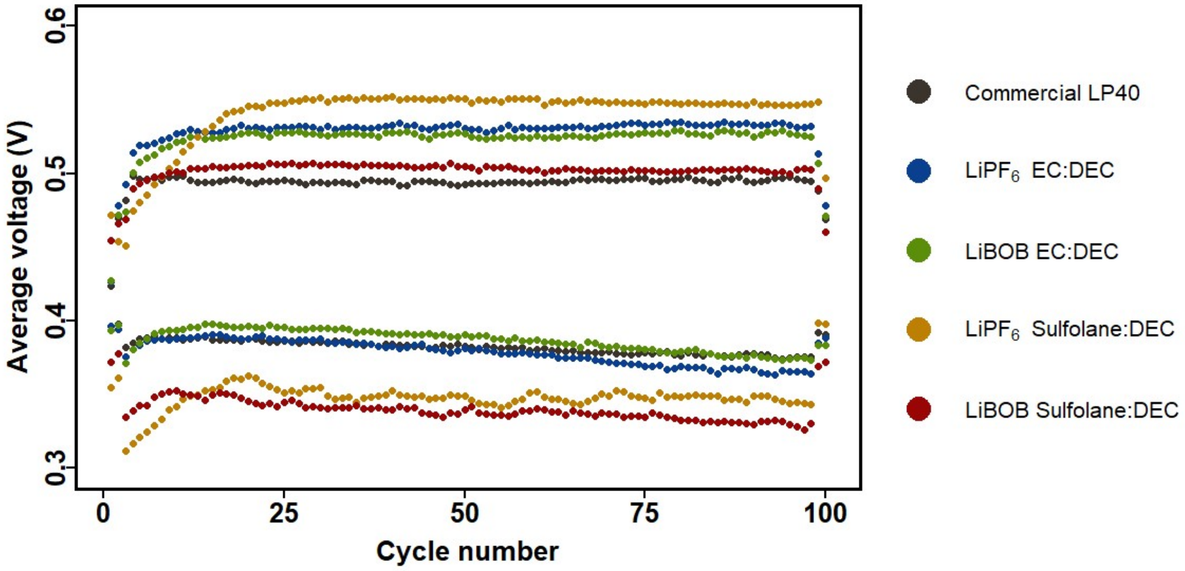

Figure S 5. Average voltage of NCA full-cells.

Table S 1. ICP-OES characterization of NCA powder show the composition of $Li_{1.021}(Ni_{0.794}Co_{0.17}Al_{0.036})O_2$.

	Sample Id	Acquisition Time	**Li 670,784 (mg/L)**	**Mn 257,610 (mg/L)**	**Co 228,616 (mg/L)**	**Ni 231,604 (mg/L)**	**Al 396,153 (mg/L)**
1	For dilution, 10% HNO_3	2021-12-20 16:08:34	0.000	0.000	0.000	0.000	0.000
2	Rerference, Multi 3 0.1 mg/L	2021-12-20 16:11:11	0.100	0.100	0.100	0.100	0.100
12	NCA_powder	2021-12-20 16:38:41	0.489	0.001	0.326	3.193	0.067
	RSD		*0.9%*	*1.4%*	*2.2%*	*3.4%*	*2.4%*
